# Social axioms on high school students in the North African context: Validation and fit of the SAS-II

**DOI:** 10.1371/journal.pone.0241510

**Published:** 2020-11-02

**Authors:** Manuel García-Alonso, Miguel Ángel Gallardo-Vigil, Patricia Melgar Alcantud, Adrián Segura-Robles

**Affiliations:** 1 Doctoral Program in Science Education, University of Granada, Melilla, Melilla, Spain; 2 Department of Research Methods and Diagnosis in Education, University of Granada, Melilla, Melilla, Spain; 3 Department of Pedagogy, University of Girona, Girona, Catalonia, Spain; 4 Department of Research Methods and Diagnosis in Education, University of Granada, Ceuta, Ceuta, Spain; Aalborg University, DENMARK

## Abstract

Social axioms or general social beliefs represent people’s cognitive map of their social world acquired through social experiences. Empirical research has related the central constructs in the study of psychology and social axioms, establishing a broad nomological network in various cultural settings. This paper studies the validity of the Social Axioms Survey II (SAS-II) short form, Spanish version, on the individual level in Melilla as North Africa´s borderland. Participants were 410 high school students from 14 to 18 years of age. The reliability analysis, the discriminant validity analysis, and the confirmatory factor analysis through the structural model equation, showed similar results to previous studies in other contexts and allowing the use of the survey in Melilla. In addition it is presented a fitted model that improves the psychometric results showing significant differences with the initial model. The confirmatory multi-group analysis of the fitted model shows measurement invariance across educational centers, allowing new research possibilities in the cultural context of Melilla.

## Introduction

Leung and Bond [1,2] argue from a functionalist perspective that social axioms or general social beliefs represent people’s cognitive map of their social world. They propose the construct of general social beliefs as an alternative and possible complement to values in interpreting culture and explaining the responses of its members, and suggested four specific functions of these general beliefs: “facilitate the attainment of important goals (instrumental), help people protect their self-worth (ego-defensive), serve as a manifestation of people’s values (value-expressive), and help people understand the world (knowledge)” (p. 288).

Social axioms are defined as generalized beliefs about people, social groups, social institutions, the physical environment, or the spiritual world, as well as categories of events and phenomena in the social world. These generalized beliefs are encoded in the form of an assertion about the relationship between two entities or concepts [1–4]. The term social refers to the assumption that axioms are acquired through social experiences and are concerned with living as inherently social beings. The term axiom refers to the assumption that these general beliefs represent basic premises that people endorse without too much scrutiny of their validity.

On the basis of a literature review, interviews and content analysis of various sources, Leung and Bond [2] developed the Social Axioms Survey, a scale of 60 items with five factors at the individual level, involving over 50 collaborators from 40 national/cultural groups. The five-factor structure, was subsequently confirmed by multilevel factor analysis technique [5].

Social axioms always involve the relationship between two conceptual entities, and the relationship may be causal or correlational. Following this approach, empirical research has related the central constructs in the study of psychology and social axioms, establishing a broad nomological network in various cultural settings [2,6–16].

The development of the Social Axioms Survey II (SAS-II) was developed using a culturally decentered approach through collaborations with psychologists from 10 countries: Brazil, China, Germany, Ghana, Israel, Japan, Malaysia, Mexico, Russia and United States [17]. The items in the later version were then consolidated, reviewed, selected and refined by a principal group of researchers, obtaining a long form of 109 items and a short form of 40 items [18].

The validation of the Social Axioms Survey II allows the research network to be extended to a multitude of cultural contexts and, therefore, to the cultural context of the city of Melilla. Melilla is a Spanish territory located in North Africa with an area of 12'32 km^2^. The National Statistics Institute in Spain registered a population of 86.487 in 2019. Due to its location, it is part of the Western Mediterranean Route used by both the Maghreb and West Africans with the intention of relocating or moving to other European countries. Irregular arrivals to Spain registered 4,984 migrants in 2019, using Melilla as the point of entry [19]. As Frank Meyer [20] describes, the religious denomination in Melilla is not only associated with questions of belief or religious practices, are mostly perceived as cultures with corresponding and clear-cut values, traditions and customs as well as a territorial rootedness. The urban societies in Melilla can be seen as good examples of the significance and interconnection of identity, culture, space and time for human co-existence and the difficult relationship of the familiar and the strange.

The particular geopolitical context and multicultural coexistence make Melilla an especially attractive enclave for social research. Therefore, the main aim of this study is guided by previous studies in other social and cultural contexts [21,22]: Validate the replication of social axioms and its five-factor structure as a representative people’s cognitive map in the cultural context of Melilla. Consequently, two specific aims were set. The first specific aim was to validate the Social Axioms Survey II (SAS-II) short form, Spanish version, on the individual level, in Melilla. The second specific aim was to develop a fitted model to improve the statistical results [18].

## Materials and methods

### Participants

The research project in which this study is framed has been developed in accordance with the ethical principles expressed in the Declaration of Helsinki. Before accessing the school-age population, the research project was firstly approved by the academic commission of the Educational Sciences Program of the University of Granada. Subsequently, it was sent to the call for institutional projects that require the participation of centers dependent on the Ministry of Education in Spain, being approved by the commission of the Vice-Dean Office for Research, International Projects and Transfer of the University of Granada. Finally, it was approved by the commission of the Ministry of Education through the Educational Programs Unit of the Provincial Department of Education in Melilla. The collaboration of the high school principals was requested through the Head of the Educational Programs Unit providing the data of the principal researcher; and in person, giving a cover letter including the research process, the ethical commitment, and a copy of the instrument to be applied. The educational centers informed the parents and legal guardians of the participants, requesting consent to participate in this research activity and keeping the informed consent in their custody. Once the consents were obtained, the centers gave the researchers a specific date and time to access the participants. In addition, the participants were previously informed by the researchers about the objectives, purpose, method, benefits of the research, their free decision to participate or not and the commitment to keep their anonymity. Finally, once the data had been analyzed, the Head of the Educational Programs Unit offered the secondary directors of the centers the possibility of obtaining the results of the investigation.

### Survey procedure and sample

The method used to select a representative sample from this population is cluster sampling. Cluster sampling does not select the subjects individually, but rather the homogeneous intergroups and heterogeneous intragroups in which they are previously inserted [23]. Clusters are represented by high schools, while the different groups available at each educational level were selected by simple random sampling. The number of students enrolled in the educational level as population (*N* = 845) was provided by the Provincial Department of Education in Melilla. Assuming a margin of error of 5%, a confidence level of 99% and a variability of 50%, a sample size of 372 subjects would have been representative enough to generalize results to the entire population. Finally, as shown in Table 1, the sample consisted of 410 students (200 males and 210 females) from 14 to 18 years of age (*M* = 15.7; *SD* = 0.915), from 6 high schools in Melilla.

**Table 1:** Characteristics of the participants.

Variable			*n*	%
**Gender**	Male		200	48.8
	Female		210	51.2
**Age**		14	34	8.3
		15	143	34.9
		16	153	37.3
		17	73	17.8
		18	7	1.7
**High School center**	High School 1		44	10.7
	High School 2		51	12.4
	High School 3		87	21.2
	High School 4		119	29.0
	High School 5		69	16.8
	High School 6		40	9.8

*N* = 410.

### Instrument

The Social Axioms Survey II (SAS-II) short form [17], Spanish version translated by Judith Gibbons, comprises 40 items, rated on a five-point Likert scale from totally disagree to totally agree. Leung et al. [17] defined the five axiom dimensions as follows: Social cynicism asserts that human nature and the social world yield negative outcomes; reward for application refers to the belief complex that people’s use of effort, knowledge, careful planning and other resources will lead to positive outcomes; social complexity asserts that people’s behavior may vary across situations and that problems have multiple solutions; fate control refers to the belief complex that life events are pre-determined by fatalistic forces, but that people may be able to predict and alter the decree of fate by various means; finally, religiosity asserts the existence of a supernatural being and the beneficial functions of religious practice.

**Fig 1:**
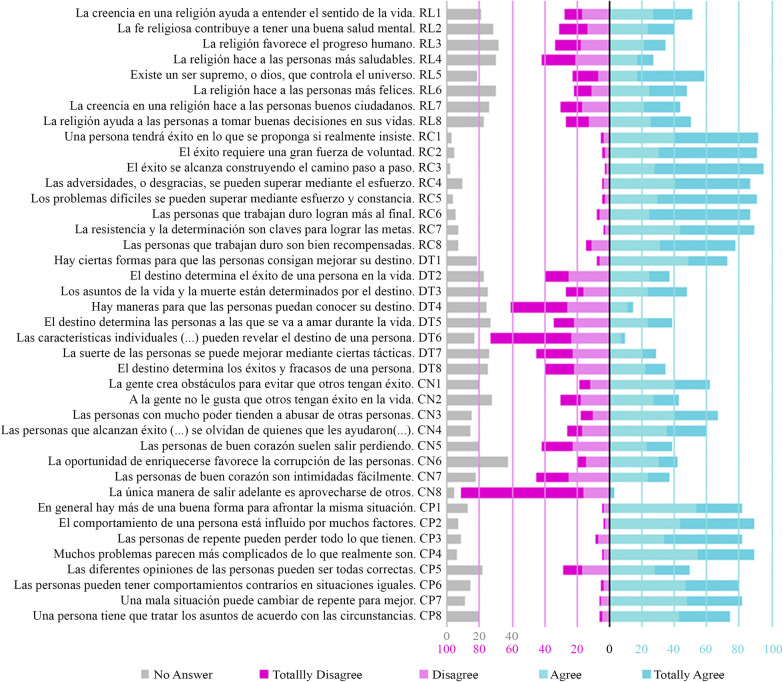
Distribution of responses to SAS-II. Items are ordered by factors: RL, Religiosity; RC, Reward for Application; DT, Fate Control; CN, Social Cynicism; CP, Social Complexity.

Distribution of responses to SAS-II is shown in Fig 1.

### Data analysis

A descriptive analysis was first performed to choose the most appropriate statistical processes. The assumption of a normal distribution of the population without prior verification makes it difficult to draw precise conclusions about reality, being a common error in a high percentage of scientific publications [24].

Some questionnaires were found to be incomplete. This is a problem since some statistical procedures do not consider the rest of the data provided by a case if it is incomplete. The elimination of cases implies the loss of present partial data, a decrease in the sample size and, therefore, a decrease in statistical power [25]. The data was missing completely at random, and the chosen method for the imputation of missing values was Multivariate Imputation by Chained Equations, using the R MICE package, a recommended choice when the data contains different types of numerical and categorical variables [26]. Missing values are imputed based on the observed values for a given individual and the observed relationships in the data for other participants, assuming that the observed variables are included in the imputation model [27].

### Psychometric analysis

The following analyses used the Lavaan package and the Semplot package for modeling structural equations in R v.3.3.2 [28]. These analyses were applied to the initial SAS-II model and a fitted model, which reported better results by deleting items DT1, DT4, DT7, CP1, CP5, CP8, CN5 and CN8.

The reliability analysis, as internal consistency, is traditionally reported using Cronbach's α [29]. Revelle and Zinbarg [30] reported the biases of this measure, encouraging researchers to use better estimators. Therefore, the reliability was analyzed by reporting Cronbach's α [29], ω_1_ [31,32], ω_2_ [33–35], and ω_3_ [36], for each factor. Following the reliability criteria [37], values above 0.90 were considered excellent, between 0.90 and 0.70 were high, between 0.70 and 0.50 were moderate, and lower than 0.50 were low.

The discriminant validity analysis indicates whether the variables are strongly uncorrelated to the factor they are measuring, allowing one factor to be distinguished from the others. The AVE, or Average Variance Extracted values of each latent construct must be greater than the maximum square correlation, AVEsqrt, with other latent construct [38].

Confirmatory factor analysis was performed using structural equation models based on covariance, using the weighted least squares mean and variance or WLSMV estimator because it is “a robust estimator that does not assume distributed variables normally and provides the best option for modeling categorical or ordered data” [39–41]. The criteria to determine an adequate fit of the models [42] are: division *χ*^2^ and degrees of freedom (*χ*^2^/*df* < 3), significance (*p* < 0.05), Comparative Fit Index (*CFI* > 0.95), Goodness of Fit Index (*GFI* > 0.9), Adjusted Goodness of Fit Index (*AGFI* > 0.9), Tucker Lewis Index (*TLI* > 0.9), Root Mean Square Residual (*RMR* < 0.05), Standardized Root Mean Square Residual (*SRMR* < 0.08), Root Mean Square Error of Approximation (*RMSEA =* 0.06–0.08).

The confirmatory analysis of multiple groups [43], using the variable schools, allowed to test the measurement invariance. The analysis of the measurement invariance proves that different groups understand the items in the same way, and are, therefore, comparable. This analysis, involves the generation of models that are evaluated in a staggered manner with greater restriction in each test: configural, metric, scalar and strict. The values Δ must be significant (*p* < 0.05) in each test.

## Results

### Distribution analysis and imputation of missing values

The following tests were performed to choose the most appropriate statistical processes: goodness-of-fit Kolmogorov-Smirnov Lilliefors (*KS* = 0.157 to 0.438; *p* < 0.001); Shapiro-Wilks (*W* = 0.94623; *p* < 0.001); multivariate asymmetry (*γˆ*^1^, *p* = 1368.164, *p* = 0); multivariate kurtosis (*γˆ*^2^, *p* = 5943.631, *p* = 0); and Levene's test (*W* = 0.011 to 3486; *p* = 0.918 to 0.063), using the sex variable to segment the sample into two independent groups. The results show that the normality assumptions are not fulfilled.

Cases with incomplete data (n = 18) show a proportion of missing values less than or equal to 4.8%. These results confirm that the decision to eliminate cases is not recommended. Little's *χ*^2^ test statistic for MCAR [27] shows that the data was missing completely at random (*χ*^2^ = 1315,572; *df* = 1275; *p* = 0.209). The Polyreg function in the R MICE package was the most suitable to impute responses in categorical variables with more than two levels, using the Bayesian polytomous regression model. The imputation of missing values increases the number of valid cases from 392 to 410.

### Reliability analysis

The reliability analysis for the initial model, shown in Table 2, report excellent values for the religiosity factor from 0.919 to 0.918; moderate values for the reward for application factor from 0.694 to 0.646, fate control factor from 0.685 to 0.652, social cynicism factor from 0.662 to 0.657; and moderate values for social complexity from 0.545 to 0.512. The fitted model reports moderately better values for each factor. The total reliability of the test allows for observing better values in the fitted model for each estimator.

**Table 2:** Reliability analysis of SAS-II and a fitted model of SAS-II.

	Model H_0_				Model H_1_			
Factors	α	ω_1_	ω_2_	ω_3_	α	ω_1_	ω_2_	ω_3_
**Religiosity**	0.919	0.919	0.919	0.918	0.919	0.919	0.919	0.918
**Reward for Application**	0.694	0.675	0.675	0.646	0.694	0.682	0.682	0.663
**Fate Control**	0.652	0.682	0.682	0.685	0.733	0.743	0.743	0.746
**Social Cynicism**	0.657	0.662	0.662	0.654	0.633	0.642	0.642	0.641
**Social Complexity**	0.545	0.530	0.530	0.512	0.561	0.547	0.547	0.528
**Total**	0.785	0.837	0.837	0.823	0.810	0.863	0.863	0.841

Model H_0_, initial model of SAS-II; Model H_1_, fitted model of SAS-II.

### Discriminant validity analysis

The discriminant validity analysis for the initial model, shown in Table 3, does not report discriminant validity for the social complexity factor being correlated with the reward for application factor (*r* = 0.384 > AVE_sqr_ = 0.362). The discriminant validity analysis for the fitted model, shown in Table 4, overcomes this problem achieving discriminant validity in all factors.

**Table 3:** Discriminant validity analysis of the initial model SAS-II.

	Correlations					AVE	AVE_sqrt_
Factors	Religiosity	Reward for Application	Fate Control	Social Cynicism	Social Complexity		
**Religiosity**	1.000	0.076	0.467	0.135	-0.136	0.586	0.766
**Reward for Application**	0.076	1.000	0.026	0.040	0.384	0.207	0.455
**Fate Control**	0.467	0.026	1.000	0.218	-0.199	0.257	0.507
**Social Cynicism**	0.135	0.040	0.218	1.000	0.246	0.216	0.465
**Social Complexity**	-0.136	0.384	-0.199	0.246	1.000	0.131	0.362

AVE, Average Variance Extracted; AVEsqrt, Square Root of Average Variance Extracted.

**Table 4:** Discriminant validity analysis of the fitted model of SAS-II.

	Correlations					AVE	AVE_sqrt_
Factors	Religiosity	Reward for Application	Fate Control	Social Cynicism	Social Complexity		
**Religiosity**	1.000	0.080	0.451	0.152	-0.079	0.586	0.766
**Reward for Application**	0.080	1.000	0.032	0.071	0.220	0.213	0.461
**Fate Control**	0.451	0.032	1.000	0.239	-0.102	0.377	0.614
**Social Cynicism**	0.152	0.071	0.239	1.000	0.389	0.242	0.492
**Social Complexity**	-0.079	0.220	-0.102	0.389	1.000	0.206	0.454

AVE, Average Variance Extracted; AVEsqrt, Square Root of Average Extracted.

### Confirmatory factor analyses

The confirmatory factor analyses, shown in Table 5, for the initial model reports a good model fit: *χ*^2^ (730) = 1254.62; *CFI* = 0.93, *TLI* = 0.92, *RMSEA* = 0.042. The fitted model also reports a good model fit with better values than the initial model: *χ*^2^ (454) = 705.69; *CFI* = 0.96, *TLI* = 0.96, *RMSEA* = 0.037.

**Table 5:** Goodness-of-fit analysis of SAS-II and a fitted model of SAS-II.

	*χ*^2^	*df*	*χ*^2^/*df*	*p*	*CFI*	*GFI*	*AGFI*	*TLI*	*RMR*	*SRMR*	*RMSEA* (C.I. 95%)
**Model H**_**0**_	1254.62	730	1.72	< 0.001	0.93	0.99	0.99	0.92	0.08	0.06	0.042 (0.04–0.05)
**Model H**_**1**_	705.69	454	1.55	< 0.001	0.96	1	1	0.96	0.07	0.06	0.037 (0.03–0.04)
**Fit criteria** ^**a**^	-	-	< 3.0	< 0.05	> 0.9	> 0.95	> 0.9	> 0.95	< 0.05	< 0.08	-

Model H_0_, initial model of SAS-II; Model H_1_, fitted model of SAS-II; *χ*^2^, Chi-Square; *df*, degrees of freedom; *p*, significant at < 0.001; *CFI*, Comparative Fit Index; *GFI*, Goodness of Fit Index; *AGFI*, Adjusted Goodness of Fit Index; *TLI*, Tucker Lewis Index, *RMR*, Root Mean Square Residual; *SRMR*, Standardized Root Mean Square Residual; *RMSEA*, Root Mean Square Error of Approximation, Confidence Interval at 95%.

^a^ Recommended criteria for an adequate fit of each model.

**Fig 2:**
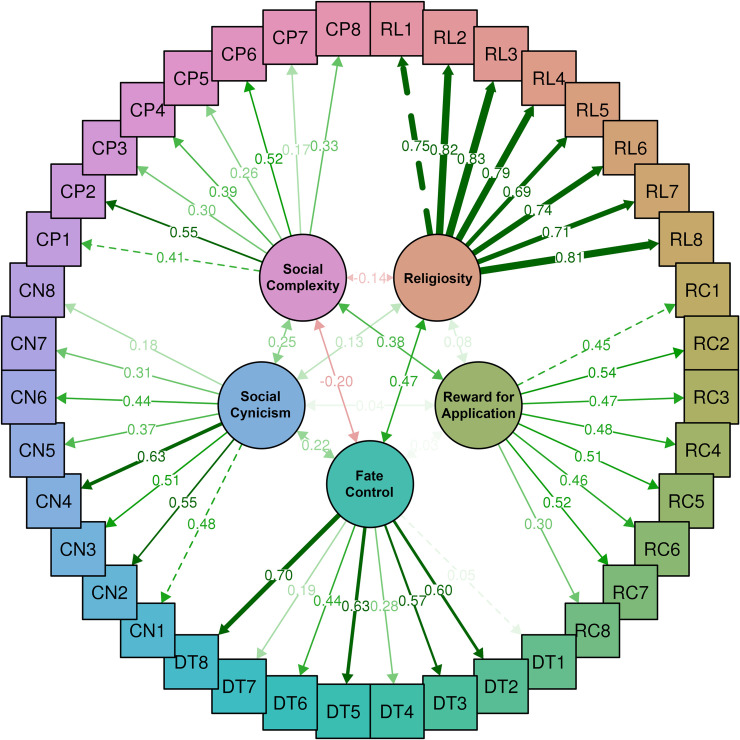
Confirmatory factor analyses for the initial model of SAS-II.

**Fig 3:**
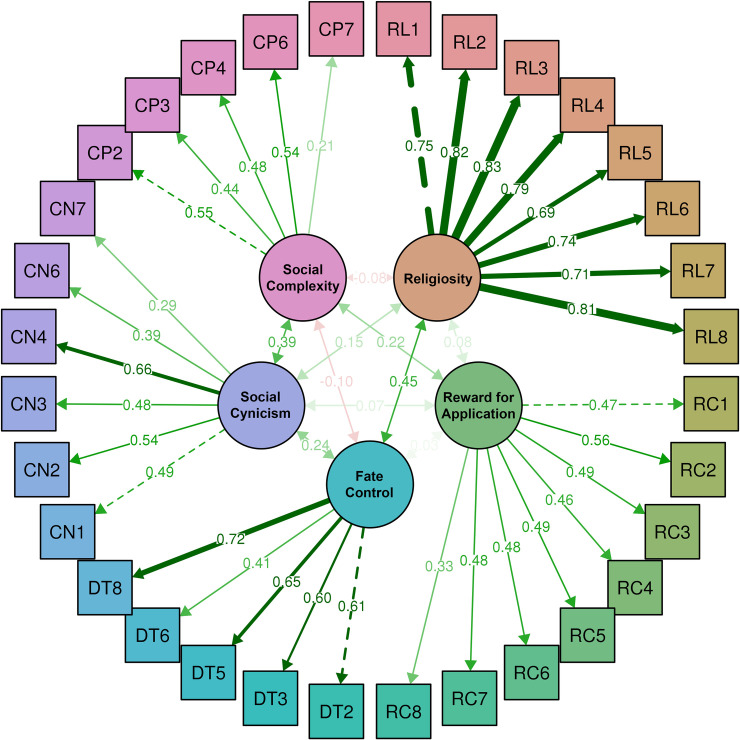
Confirmatory factor analyses for the fitted model of SAS-II.

The loadings of each item to its corresponding factor for the initial model is shown in Fig 2, and the loadings of each item to its corresponding factor for the fitted model is shown in Fig 3.

The difference test for the initial model and fitted model, shown in Table 6, reports significant differences (*p* < 0.001).

**Table 6:** Difference test of SAS-II and a fitted model of SAS-II.

	*χ*^2^_dif_	*df*_dif_	*p*
**Model H**_**0**_	-	-	-
**Model H**_**1**_	548.93	276	< 0.001

Model H_0_, initial model of SAS-II; Model H_1_, fitted model of SAS-II; *χ*^2^_dif_, difference of Chi-Square from previous model; *df*_dif_, difference of degrees of freedom from previous model.

### Measurement of invariance

The measurement invariance test, shown in Table 7, supports that the instrument measures the same psychological construct in all educational centers. Holding this assumption, the comparisons between groups are valid and can be meaningfully interpreted.

**Table 7:** Measurement invariance test across educational centers of a fitted model of SAS-II.

Model	*df*	*AIC*	*BIC*	*χ*^2^	*χ*^2^_dif_	*df*_dif_	*p*	*CFI*	*RMSEA*	Δ*CFI*	Δ*RMSEA*
**Model**_**1**_	2724	35942	38496	4005.5	-	-	-	0.703	0.083	-	-
**Model**_**2**_	2859	35944	37956	4277.5	271.97	135	< 0.001	0.671	0.085	0.032	0.002
**Model**_**3**_	2994	35848	37318	4451.9	174.35	135	< 0.012	0.662	0.084	0.009	0.001
**Model**_**4**_	3019	35938	37307	4591.2	139.34	25	< 0.001	0.635	0.087	0.027	0.003

*df*, degrees of freedom; *AIC*, Akaike Information Criterion; *BIC*, Bayesian Information Criterion; *χ*^2^, Chi-Square; *CFI*, Comparative Fit Index; *RMSEA*, Root Mean Square Error of Approximation; Δ, change in fit indices from previous model.

## Discussion

The main aim of this study was to validate the replication of social axioms and its five-factor structure as a representative people’s cognitive map in the cultural context of Melilla for the first time, as one of the two European Union’s land borders with North Africa. This geographical, political and multicultural situation provides its own social characteristics that cannot be directly attributed to the nations of Spain or Morocco. The second aim was to develop a fitted model to improve the psychometrical measures of the Social Axioms Survey II (SAS-II).

The reliability analysis for the initial model, shown in Table 2, reported moderate values for social complexity from 0.545 to 0.512. The fitted model reports moderately better values for each factor. Social Complexity and Fate Control had shown marginal reliability as some previous researches [17]. The Social Complexity factor thus remains problematic; this is the same factor from the original social axioms survey that Barnard et al. [4] were unable to replicate in their South African study.

The discriminant validity problem for the social complexity factor was overcome for the fitted model, as shown in Tables 3 and 4, achieving discriminant validity in all factors.

Confirmatory factor analyses through the structural model equation, shown in Fig 2, report low item loads at their corresponding factor for both models. However, goodness-of-fit estimators report good values for both models, as shown in Table 6, reporting better values for the fitted model.

The measurement invariance test, shown in Table 7, supports that the comparisons between groups are valid and can be meaningfully interpreted. This suggests that the fitted SAS-II model could be a valid instrument to study the differences or similarities between educational centers as microcultural contexts.

## Conclusions

Considering the results of the analysis, we can affirm that the first objective has been achieved moderately, finding problems of discriminant validity for the factor of social complexity. Likewise, we can affirm that the second objective has been achieved, obtaining a valid model to investigate social axioms in the cultural context of Melilla. The findings of this study provide possibilities for expanding the nomological network of correlations in the field of social psychology and invite the study of possible causal relationships from these investigations.

## Limitations of the study

This study is limited by access to the population. Two institutes did not respond to our request for participation in this research, one of them characterized by students of Sephardic culture as a representative cultural identity of the city, which could have influenced the results of the survey. In addition, as has been shown in previous research, the social complexity factor showed lower values than the other factors, therefore, this factor should be approached with caution in future research.
